# Draft Genome Sequence of *Saccharomycopsis fermentans* CBS 7830, a Predacious Yeast Belonging to the *Saccharomycetales*

**DOI:** 10.1128/genomeA.01445-17

**Published:** 2018-01-11

**Authors:** Ana Hesselbart, Klara Junker, Jürgen Wendland

**Affiliations:** a Carlsberg Research Laboratory, Yeast & Fermentation, Copenhagen, Denmark; b Vrije Universiteit Brussel, Functional Yeast Genomics, Brussels, Belgium

## Abstract

*Saccharomycopsis fermentans* is an ascomycetous necrotrophic fungal pathogen that penetrates and kills fungal prey cells via targeted penetration pegs. Here, we report the draft genome sequence and scaffold assembly of this mycoparasite.

## GENOME ANNOUNCEMENT

*Saccharomycopsis* is the sole genus of the family *Saccharomycopsidaceae* (1). One member of this genus, the starch-degrading yeast *S. fibuligera*, is known for its contributions to rice wine fermentation (2). The role of *S. fibuligera* as an enzyme producer also spurred interest in using this yeast for bioethanol production (3). Recently, whole-genome sequencing studies revealed that the genome of *S. fibuligera* encompasses seven chromosomes with about 18 Mb (4). Other members of the genus *Saccharomycopsis*, including *S. fermentans*, have been described as predacious yeasts that generate penetration pegs with which they kill other fungal cells utilizing prey cell content (5). *S. fermentans* (formerly classified as *Arthroascus fermentans*) was isolated in 1994 from the soil of a Taiwanese orchard and was shown to ferment glucose (references 6 and 7 and references therein). *Saccharomycopsis* species are natural auxotrophs for organic sulfur (8). In *S. fibuligera*, the absence of genes involved in sulfate assimilation has been observed (4).

The usefulness of non-*S. cerevisiae* yeasts in fermentation and biotechnology depends on detailed characterization of the microbial genomes. Thus, additional draft genome sequences are required to not only provide species-specific markers for identification but also initiate pathway analyses and enable targeted strain improvements. Furthermore, a deeper understanding of the molecular biology of *Saccharomycopsis* species—specifically of their predacious behavior—is warranted. To this end, we recently established the draft genome of another predacious *Saccharomycopsis* yeast, *S. fodiens* strain CBS 8332 (9). We confirmed that, just like *S. fibuligera*, *S. fodiens* also lacks the genes in the sulfate assimilation pathway.

Here, we report the draft genome sequence of *S. fermentans*, obtained using Illumina MiSeq paired-end read sequencing. *S. fermentans* was grown in complex medium (1% [wt/vol] yeast extract-peptone-dextrose [YPD], 2% [wt/vol] peptone, and 2% [wt/vol] dextrose) at 30°C with constant shaking. DNA extraction and sequencing were performed at LGC Genomics (Berlin, Germany). Two paired-end libraries were sequenced, producing 10,363,062 raw reads. These were quality trimmed, resulting in 4,807,796 high-quality reads with the short fragment library and 4,521,208 high-quality reads with an 8-kb mate pair library. These were assembled using Bowtie2 version 2.1.0. The initial assembly generated 160 contigs with a total content of 14,266,439 bp and a contig *N*_50_ of 265,725 bp. The contigs were then further assembled into 33 scaffolds harboring 14,461,413 bp, with an *N*_50_ of 2,146,288 bp. The longest scaffold contains 3,513,907 bp, with 13 scaffolds larger than 20 kb. The overall GC content is 35.1%.

For a draft annotation of the nuclear genome, open reading frames (ORFs) with a size of >300 nt were predicted and compared using blastx to translated proteins in the *Saccharomyces cerevisiae* genome. In all, 5,917 nonoverlapping ORFs were detected in *S. fermentans*, and of those, 3,882 genes produced hits with *S. cerevisiae*. An additional blast search against other organisms in the nonredundant database at NCBI (https://blast.ncbi.nlm.nih.gov/Blast.cgi) generated a further 263 hits (E values, <10^−10^). We could also verify the absence of genes required for sulfate assimilation in *S. fermentans*. Additionally, we identified 149 tRNA genes in the *S. fermentans* genome using tRNAscan-SE (10).

### Accession number(s).

This whole-genome shotgun project has been deposited in DDBJ/ENA/GenBank under the accession no. JNFW00000000. The version described in this paper is the first version, JNFW01000000.
